# Extracting extensor digitorum communis activation patterns using high-density surface electromyography

**DOI:** 10.3389/fphys.2015.00279

**Published:** 2015-10-06

**Authors:** Xiaogang Hu, Nina L. Suresh, Cindy Xue, William Z. Rymer

**Affiliations:** ^1^Sensory Motor Performance Program, Single Motor Unit Lab, Rehabilitation Institute of ChicagoChicago, IL, USA; ^2^Department of Biomedical Engineering, Chinese University of Hong KongHong Kong, China; ^3^Department of Physical Medicine and Rehabilitation, Feinberg School of Medicine, Northwestern UniversityChicago, IL, USA

**Keywords:** finger extension, finger individuation, muscle compartment, extensor activation, HD EMG

## Abstract

The extensor digitorum communis muscle plays an important role in hand dexterity during object manipulations. This multi-tendinous muscle is believed to be controlled through separate motoneuron pools, thereby forming different compartments that control individual digits. However, due to the complex anatomical variations across individuals and the flexibility of neural control strategies, the spatial activation patterns of the extensor digitorum communis compartments during individual finger extension have not been fully tracked under different task conditions. The objective of this study was to quantify the global spatial activation patterns of the extensor digitorum communis using high-density (7 × 9) surface electromyogram (EMG) recordings. The muscle activation map (based on the root mean square of the EMG) was constructed when subjects performed individual four finger extensions at the metacarpophalangeal joint, at different effort levels and under different finger constraints (static and dynamic). Our results revealed distinct activation patterns during individual finger extensions, especially between index and middle finger extensions, although the activation between ring and little finger extensions showed strong covariance. The activation map was relatively consistent at different muscle contraction levels and for different finger constraint conditions. We also found that distinct activation patterns were more discernible in the proximal–distal direction than in the radial–ulnar direction. The global spatial activation map utilizing surface grid EMG of the extensor digitorum communis muscle provides information for localizing individual compartments of the extensor muscle during finger extensions. This is of potential value for identifying more selective control input for assistive devices. Such information can also provide a basis for understanding hand impairment in individuals with neural disorders.

## Introduction

The convergent and divergent spinal neuronal networks and the multi-tendinous extrinsic finger muscles afford the dexterous control of human finger movement. Although some level of independent control is needed, individual fingers cannot move completely independently (Kilbreath and Gandevia, 1994; Zatsiorsky et al., 1998; Häger-Ross and Schieber, 2000; Schieber and Santello, 2004), especially in populations with neurological disorders (Lang and Schieber, 2003; Lee et al., 2013). These coupled finger movements can arise from mechanical coupling and/or from non-selective neural control. The mechanical coupling arises from the passive tissue connections between tendons of the hand, and tendon re-branching between digits (Malerich et al., 1987; von Schroeder et al., 1990; Leijnse et al., 1997). The divergent projections of cortical neurons to multiple motoneuron pools across compartments result in synchronized activation of motoneurons innervating multiple compartments of the finger muscles (Zatsiorsky et al., 2000; Keen and Fuglevand, 2004a,b). These complex anatomical structures and neural control strategies impose challenges for quantifying the extensor muscle spatial activation patterns under different task requirements.

The localized activation of the extensor digitorum communis has been examined through the analysis of motor unit recruitment (van Duinen et al., 2009) and through the analysis of discharge synchronization (Keen and Fuglevand, 2004a,b) using focal intramuscular electromyogram (EMG) recordings. However, the overall spatial activation pattern of the extensor digitorum communis muscle during individual finger extension has not been fully characterized, because locating these compartments solely based on the anatomy is problematic. In particular, the extensor tendinous structures vary across individuals (Zilber and Oberlin, 2004), and considerable cross-talk is inevitable even with careful placement of individual surface EMG electrodes (Leijnse et al., 2008a). Using high density surface EMG recording techniques, a recent study has quantified the spatial distributions of dorsal forearm muscles including wrist extensors, wrist radial/ulnar deviators, and finger extensors (Gallina and Botter, 2013). However, due to a limitation of the recording area of the grid electrodes, only a portion of the extensor digitorum communis muscle was recorded, in that only middle, ring, and little finger extensions were examined. In addition, only isometric extensions were tested, and different contraction conditions, such as dynamic contractions (length of the muscle-tendon unit allowed to change), may induce different degrees of shift in the spatial distribution of the muscle activation.

Accordingly, the purpose of the current study was to quantitatively map the global activation patterns of the entire extensor digitorum communis using high-density (HD) surface EMG recordings during different muscle contraction conditions. The muscle activation map based on the root mean square (RMS) of the EMG obtained from a 7 × 9 electrode grid was constructed when subjects performed individual finger extensions of all four fingers of the hand, at different effort levels and at different finger constraint conditions. The different effort levels were tested to examine whether the activation patterns change with progressive recruitment of the muscle. At the different finger constraint conditions, the finger was either allowed to move freely (i.e., dynamic extension) or was constrained (i.e., isometric extension), in order to test the consistency of the activation map when the muscle belly was shortened, or was largely constrained. The global spatial activation map of the extensor digitorum communis provides information for localizing the active compartments of the muscle. The HD EMG electrode grid allows us to track the spatial activation (often varying) at different tasks and over different individuals, without a priori explicit knowledge of the specific active region of the muscle compartments. The findings also provide a basis for understanding the impairment (i.e., reduced/loss of individualized finger control) of hand function in clinical populations such as in stroke survivors.

## Materials and methods

### Participants

Ten right-dominant neurologically intact individuals (four male, six female) volunteered to participate in this study. The EMG activity of the extensor digitorum communis muscle was examined during static and dynamic extension of individual fingers at different effort levels. All participants gave informed consent via protocols approved by the Institutional Review Board under the Office for the Protection of Human Subjects at Northwestern University.

### Procedures

**Figure 1:**
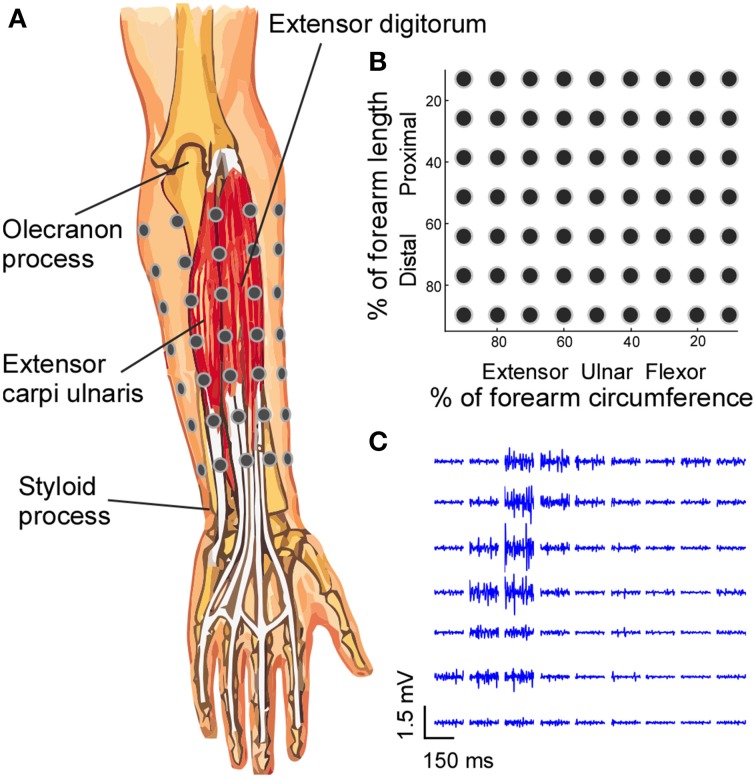
**Electrode placement and EMG signals. (A)** A 7 × 9 EMG electrode grid was placed over the skin of the forearm based on the anatomical landmarks of the forearm, and the absolute inter-electrode distance was not uniform. **(B)** The grid organization is presented in the relative forearm length and circumference dimensions. **(C)** The segments (150 ms) of EMG signals recorded from all electrodes during a four-finger extension task are shown.

Participants were seated upright in a chair with their forearm pronated 90° resting on a table and wrist in 0° (radial or ulnar) deviation. Their palm rested on an aluminum 120° arc plate, resulting the wrist in 30° extension and fingers in 60° flexion about the metacarpophalangeal joint and 0° flexion or extension about the interphalangeal joints. During the experiment, subjects were asked to extend their individual metacarpophalangeal joints either freely (dynamic condition) or while their fingers were constrained by a plastic board (static condition). In both dynamic and static conditions, the increment of muscle contraction level was self-paced at a comfortable rate for individual subjects. In dynamic conditions, subjects either performed a full extension (~60°) termed high effort, or they extended their joint (~30°) until their digits were horizontal in parallel with the table surface, termed low effort. In static conditions, they extended their fingers against the plastic board using high or low efforts, and visual feedback of the EMG signals from all the channels were provided to guide their effort level. The subjects also extended all their four fingers simultaneously as a control (dynamic or static and high or low efforts) for each condition. The effort level was quantified using the average RMS of EMG signals recorded across all the channels. At each condition, subjects were instructed to extend their fingers and maintain the steady effort for 5 s, and they repeated the task three times with a 4 s of relaxation period between contractions (Figure 1). Prior to the main testing session, subjects were given practice trials to become familiarized with the task to ensure that they can perform the individual finger extension at different effort levels. The different conditions were randomized within and between subjects.

EMG activity was recorded from the forearm muscles, with the surface grid electrode centered on the extensor digitorum communis muscle. Activity from the surrounding muscles and flexor muscles were also recorded to monitor possible signal contamination and muscle co-contractions. To standardize the placement of electrodes, the forearm was divided into seven equally-spaced segments from the olecranon process to the styloid process of the ulna. Prior to the electrode placement, the skin of the forearm was shaved and cleaned with abrasive alcohol pad (70% alcohol + pumice) and then with regular alcohol pad (70% alcohol) to ensure high signal quality. At the proximal–distal center of each segment, a row of nine surface EMG electrodes (monopolar electrodes with 1 mm diameter recording surface and 1.5 m long shielded cables, TMSi) were placed on the skin with equal inter-electrode distance for each row (Figure 1). The relatively small 1 mm diameter recording surface reduces the recording area and the degree of cross-talk compared with the regular centimeter-scale surface electrode pad (Helal and Bouissou, 1992; Farina and Merletti, 2001).

Because the circumference of the forearm decreases from the proximal to distal end, the inter-electrode distance varied between rows. The position of the electrodes for each row was secured using an elastic band with equal inter-electrode spacing, to ensure that the electrodes covered the entire circumference of the forearm and that the electrode–skin contact was secure. With a larger circumference, the elastic band was stretched more to cover the forearm circumference, and *vice versa*. The reference electrode was placed over the lateral epicondyle at the elbow joint. The 63 monopolar EMG signals were amplified (Rafa, TMSi) with a band-pass filter of 5–1000 Hz and the data were sampled at 2 kHz.

### Data analysis

EMG signals were off-line band-pass filtered (second order Butterworth, double pass, 20–400 Hz). Given that the inter-electrode distance was not uniform across all recorded channels, monopolar EMG signals were used for further analysis. To characterize the spatial distribution of the muscle activation, the root mean squared (RMS) values of the 63 channels were calculated in a 7 × 9 grid for each condition, and the average of the three repetitions were used to obtain the RMS map. All the RMS maps were constructed based on the relative dimensions (i.e., the % of forearm length in vertical axis and % of forearm circumference in the horizontal axis). The RMS maps were interpolated linearly six times in the relative dimensions. The interpolation was performed just for visual presentations, and all the later centroid and SSD calculations was based on the original un-interpolated data. EMG signals during the 5-s steady state contraction were analyzed for the RMS calculation. To reduce the influence of background noise and spontaneous muscle activity during resting state, the RMS-values of all the EMG channels during active state were subtracted from RMS-value during 2 s of the resting state. The X (% of forearm circumference) and Y (% of proximal–distal forearm length) coordinates of the RMS grid map were calculated to quantify the activation distribution (Equations 1 and 2). The Y coordinates were calculated as a percentage from the proximal to distal landmarks (0% at the olecranon process and 100% at the styloid process of the ulna) of the forearm. The X coordinates were calculated as a percentage of the forearm circumference (0% on the medial side of the forearm to flexor, then to extensor, and lastly to 100% back on the medial side of the forearm.

(1) $$C_{x}=\frac{\sum_{i}\sum_{j}\left(RMS_{ij}\cdot \;x_{i}\right)}{\sum_{i}\sum_{j}\left(RMS_{ij}\right)}$$

(2) $$C_{y}=\frac{\sum_{i}\sum_{j}\left(RMS_{ij}\cdot \;y_{j}\right)}{\sum_{i}\sum_{j}\left(RMS_{ij}\right)}$$

Here, *C*_*X*_ and *C*_*y*_ represent the centroid coordinates in the X and Y directions, *RMS*_*ij*_ represent the *i* × *j*th element in the 7 × 9 RMS grid, and *x*_*i*_ and *y*_*j*_ represent the *x* and *y* coordinates (relative dimensions) of the *i* × *j*th element.

Given the compartmentalization of the extensor digitorum communis, the computed centroid may not exhibit enough resolution to identify specific shift of activation distributions, because the centroid only gives the center of the distribution, and different distribution shapes can give rise to identical centroid. Alternatively, we calculated the sum of squared difference (SSD) of the normalized RMS between the different conditions (four-finger extension vs. individual finger extension, dynamic vs. static conditions, and high vs. low contraction conditions; Equation 3). The SSD between individual finger and four-finger extensions was calculated to estimate the difference in EDC spatial activation patterns between single finger and all four-finger extensions. The SSD (dynamic vs. static conditions) was calculated to test potential EDC activation differences in these two different finger constraint conditions. The SSD (high vs. low contractions) was calculated to estimate the difference of activation at different muscle contraction levels.

(3) $$SSD=\frac{\sum_{i}\sum_{j}{\left(nRMS_{A}-nRMS_{B}\right)}^{2}}{\sum_{i}\sum_{j}{\left(nRMS_{A}-nRMS_{B}\right)}^{2}}\;\cdot \;100\%$$

Here, *SSD* represents the sum of squared difference in the normalized RMS grid (with the maximum element normalized to one). RMS maps were normalized with respect to their own maximum values. If two normalized RMS maps are identical, the SSD would be zero. The SSD increases as the two RMS maps start to differ, and two random RMS grids lead to 100, *nRMS*_*A*_ represents the normalized RMS grid in four-finger extension, dynamic, or high contraction conditions, and *nRMS*_*B*_ represents the normalized RMS in individual finger extension, static, or low contraction conditions. The SSD calculation can capture the differences in specific spatial distribution, especially the shape of distribution. Thus, the SSD index is more sensitive than the centroid index in quantifying the changes in specific activation shapes.

Regarding the centroid and SSD calculations, we have included the RMS of all the 63 channels, which can take into account undesired low level co-activation of other muscles or muscle compartments. This all inclusive approach can also accurately estimate the overall level of muscle activation, provided that a very large number of channels are not used. However, when a very limited number of channels register EMG activities out of a large number of grid channels, the estimated activation level could be minimal due to an offset of a large number of inactive channels. Alternatively, one can select the “active” channels (e.g., using a cluster analysis; Vieira et al., 2010), and just focus on the regional muscle activity with high level activations. This channel segmentation approach can limit the influence of inactive channels (suitable for a very large number of grid channels) or the channels that registered low level muscle activations. However, one purpose of our current study was to investigate the influence of potentially low level co-activations of other muscles and/or muscle compartments. We placed electrodes over the entire forearm, covering both extensors and flexors, in order to track the influence of undesired muscle activations on the estimation of individuated extensor digitorum communis compartment activation, and therefore, the EMG activities of all the channels were used for the calculation.

#### Statistical analysis

Assuming a normal distribution, a repeated measures Three-way [finger constraint (two levels) × effort level (two levels) × finger involvement (five levels)] analysis of variance (ANOVA) was performed on the mean RMS-values in different finger constraint conditions and different levels of muscle activation using different fingers, with null hypothesis that no difference was found in RMS-values across these different conditions. Mauchly's-test was used to test sphericity (homogeneity of variances), and a Greenhouse–Geisser correction was used to modify the degrees of freedom, when the sphericity test was significant. When necessary, *post hoc* pairwise multiple comparisons with Tukey's correction method were used. The same Three-way ANOVA procedure was performed on the X and Y coordinates of the centroids and the SSD between the four-finger extension task and the individual finger extension tasks. Lastly, a repeated measures Two-way ANOVA was performed on the SSD-values between dynamic and static conditions [effort level (two levels) × finger involvement (five levels), **Figure 5B**] as well as between high and low effort conditions [finger constraint (two levels) × finger involvement (five levels), **Figure 5C**] during different individual finger extension tasks. *Post hoc* pairwise multiple comparisons with Tukey's correction method were used, when the main effect was significant. A significant *p*-value smaller than 0.05 was used in the test.

## Results

### Muscle activation level

**Figure 2:**
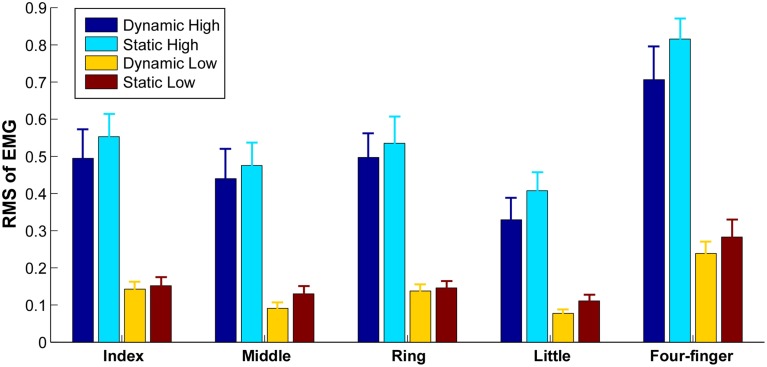
**Average RMS within each grid in different tasks**. Error bars represent standard errors between subjects.

The levels of extensor digitorum communis activation estimated from the mean RMS-values across 63 channels are illustrated in Figure 2. The highest mean RMS-value (during four-finger extension at high effort) within a subject was used to normalize the mean RMS-value, because the RMS-value varies considerably across subjects. As expected, the muscle activation level was significantly higher during high effort finger extension than during low effort conditions [*F*_(1, 9)_ = 34.91, *p* = 0.001]. No significant interactions (*p* > 0.05) were found between *finger* (individual fingers vs. whole hand), *effort level* (high vs. low), and *finger-constraint* (dynamic vs. static). A significant difference in the RMS at different finger tasks was evident [*F*_(4, 36)_ = 29.26, *p* = 0.001]. The *post hoc* analysis showed that the RMS was higher in four-finger task compared with all the individual finger tasks (*p* < 0.05), and that the RMS in little finger extension was lower than the RMS in index and ring finger extension tasks (*p* < 0.05). The activation level was not significantly different between the dynamic and static extension tasks [*F*_(1, 9)_ = 0.24, *p* = 0.64].

### Extensor digitorum communis activation distribution

**Figure 3:**
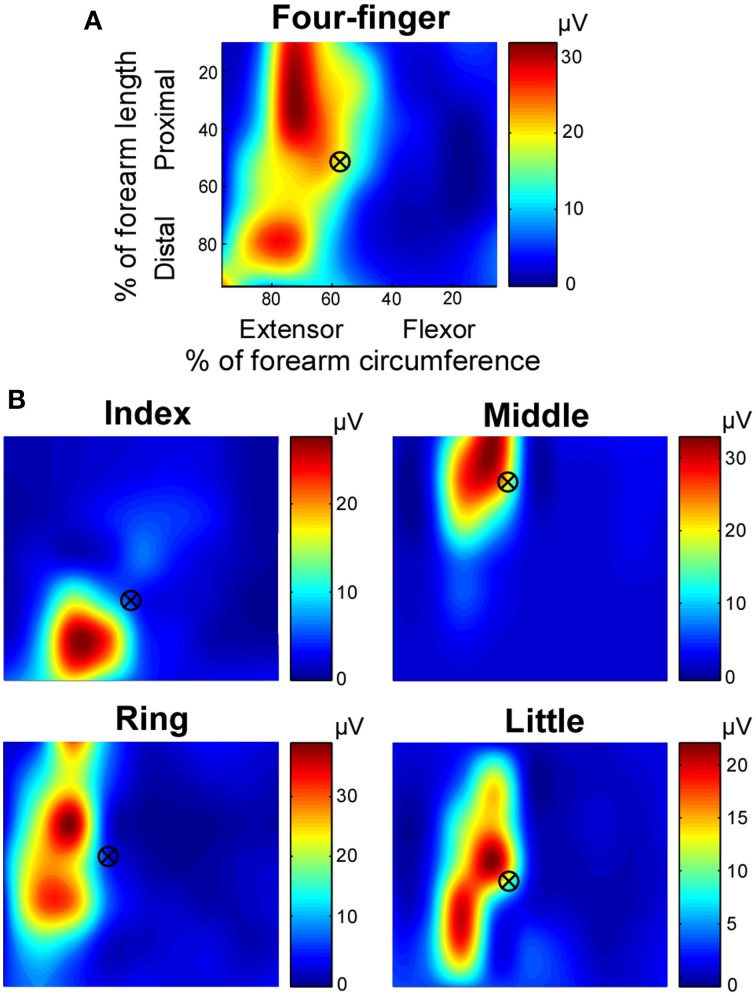
**Exemplar root mean square (RMS) map of individual finger and four-finger extensions**. The RMS maps, based on monopolar EMG signals, were shown in relative dimensions. **(A)** RMS map in the four-finger extension task. **(B)** RMS maps in individual finger extension tasks. The centroid marks are also shown over the RMS map. Note that the color coding scales individually with each map.

The exemplar distributions of muscle activation when different fingers extended in dynamic and high effort conditions are shown in Figure 3. The centroids of the RMS are shown in crossed circles. When the four fingers extended simultaneously (Figure 3A), the entire extensor digitorum communis was active. However, when individual fingers extended separately (Figure 3B), distinct regions of the extensor digitorum were selectively activated, with the index finger in the most distal region, the middle finger in the most proximal region, and the ring and little fingers in between. Such distinct regions of activation allow the detection of anatomical locations of extensor digitorum in controlling individual finger extensions.

**Figure 4:**
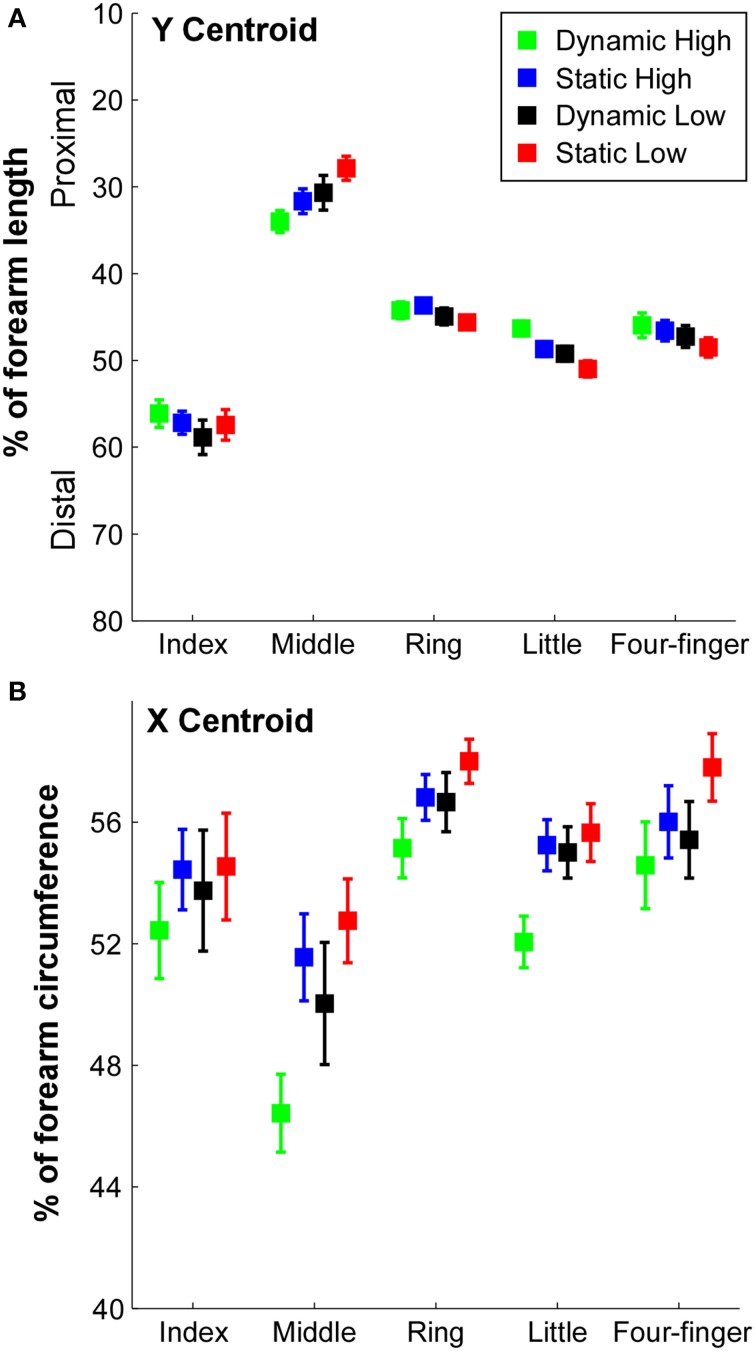
**X and Y centroid locations of the RMS in different tasks. (A)** The Y coordinate of the centroid in the longitudinal direction was calculated as a percentage of the forearm length from the olecranon process (0%) to the styloid process (100%) of the ulna. Error bars represent standard errors between subjects. **(B)** The X coordinate of the centroid in the circumferential direction was calculated as a percentage of forearm circumference from radial–flexor to ulnar–extensor (0% on the medial side of the forearm to flexor, then to extensor, and to 100% on the medial side of the forearm).

The locations of the centroid at different task conditions are illustrated in Figure 4. The Y coordinate of the centroid (Figure 4A) revealed that the centroid of the index finger was close to 60% from the olecranon process, whereas the centroid of the middle finger was ~30% from the olecranon process. The centroids of the ring and little fingers were 40–50% from the olecranon process. With a high contraction effort and dynamic contraction conditions, the centroids in the ring and little finger extension as well as the four-finger extension tended to shift toward the proximal end, whereas the centroid of the middle finger extension tended to shift toward the distal end. The Two-way ANOVA showed a significant *finger* × *effort level* interaction [*F*_(4, 36)_ = 6.03, *p* = 0.001] and *finger* × *constraint* interaction [*F*_(4, 36)_ = 2.93, *p* = 0.034]. The centroids of the index finger were significantly lower (more distal) than the rest of the fingers (*p* < 0.05), and the centroids of the middle finger were significantly higher (more proximal) than the rest of the fingers (*p* < 0.05). The centroids of the ring finger in the static conditions and dynamic low condition were higher than the centroids of the little finger (*p* < 0.05).

The X coordinate of the centroids are illustrated in Figure 4B. A significant difference of centroids between fingers was evident [*F*_(4, 36)_ = 16.49, *p* = 0.001] and a significant *effort level* × *constraint* interaction [*F*_(1, 9)_ = 19.10, *p* = 0.002] was also found. The centroid of the middle finger was closer to the ulnar side compared with other fingers (*p* < 0.05). The centroid of the ring finger was closer to the radial side than other fingers (*p* < 0.05). In individual finger extension tasks, the centroid during dynamic high effort contraction was closer to the ulnar side compared with other tasks (*p* < 0.05), and the centroids during static low effort contraction of the middle and ring fingers were closer to the radial side compared with other tasks (*p* < 0.05).

### Activation differences between task conditions

**Figure 5:**
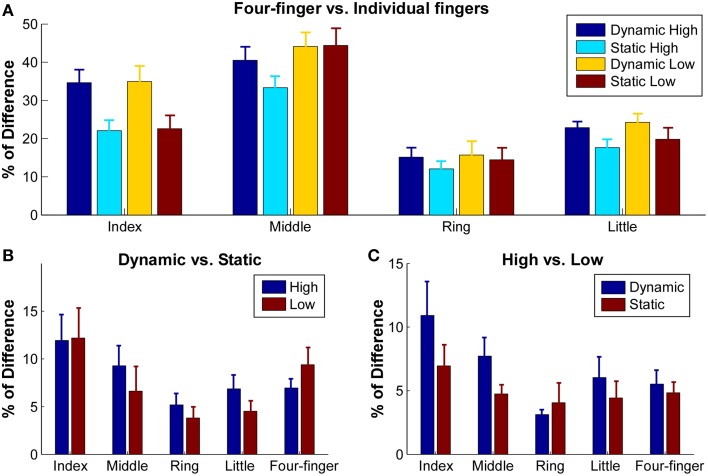
**Sum of squared difference (SSD) between normalized RMS maps of different tasks**. RMS maps were normalized with respect to their own maximum values. **(A)** SSD between four-finger extension and individual finger extensions in different conditions. Error bars represent standard errors between subjects. **(B)** SSD between dynamic and static extensions in high and low conditions. **(C)** SSD between high and low extensions in dynamic and static conditions.

Given the inhomogeneity of the extensor digitorum communis activation, the centroid may not be sensitive enough to identify specific shift of activation distribution shapes. To better quantify the difference of activation, we calculated the sum of squared difference (SSD) in the normalized RMS between the different task conditions (Figure 5). When the SSD between the four-finger extension and individual finger extension was calculated to estimate the difference in extensor digitorum communis spatial activation patterns between single finger and all four-finger extensions (Figure 5A), ~22–36% of difference was found in index finger extension, and ~40% of difference was found in middle finger extension. In contrast, only about 12–23% of difference was found in ring and little finger extensions. The ANOVA revealed a significant *finger* × *constraint* interaction [*F*_(3, 27)_ = 9.20, *p* = 0.001], and the SSD was not influenced by effort level [*F*_(1, 9)_ = 1.11, *p* = 0.32]. In index and little finger extension tasks, the SSD was significantly larger in the dynamic than the static contractions (*p* < 0.05).

The influence of finger constraints (dynamic vs. static) on the activation patterns was further examined within the finger extension tasks to test potential extensor digitorum communis activation differences in these two different finger constraint conditions (Figure 5B). The SSD between the dynamic and static contractions ranged from 5.2 to 11.6%. A significant effect was found between individual fingers [*F*_(4, 36)_ = 2.64, *p* = 0.049]. The SSD in index extension was significantly higher than ring extension (*p* < 0.05). However, the SSD was not influenced by the effort level [*F*_(1, 9)_ = 2.78, *p* = 0.13].

The influence of effort levels on activation patterns was also examined within the finger extension tasks to estimate the difference of activation at different muscle contraction levels (Figure 5C). The SSD between the low and high effort contractions ranged from 4.4 to 10.9%. A significant effect was found between individual fingers [*F*_(4, 36)_ = 3.70, *p* = 0.013]. The SSD in index extension was significantly higher than ring, little finger and four-finger extensions (*p* < 0.05). The SSD did not differ at the two different constraint conditions [*F*_(1, 9)_ = 0.036, *p* = 0.86].

## Discussion

The purpose of this study was to geometrically quantify the global spatial activation patterns from recorded HD surface EMG of the extensor digitorum communis during individuated finger extensions at different task conditions. We found that the computed activation map of the extensor digitorum communis was most distinct during index and middle finger extensions; however, the regions of activation were not well-separated during ring and little finger extensions. The spatial activation map was relatively consistent across different muscle contraction levels and at different finger constraint conditions. Consistent with an earlier study (Gallina and Botter, 2013), we also found that the distinct activation patterns were better separated in the proximal–distal direction than in the radial–ulnar direction, partly due to the anatomical orientation of the extensor muscle. Overall, our computed spatial activation maps of the extensor muscle provides information to identify local active regions of the extensor digitorum communis muscle during finger extensions.

### Selective activation of extensor muscle compartments

Both mechanical and neural enslaving effects limit the individualized finger movement. For example, the juncturae tendinum, narrow bands of passive tissue, connects the four extensor digitorum communis tendons, which redistribute the extensor muscle forces and limit individualized finger motion (von Schroeder et al., 1990; Keen and Fuglevand, 2003). The common inputs to different compartments of the extensor muscle also extend multiple fingers simultaneously (Keen and Fuglevand, 2004a,b; van Duinen et al., 2009). These enslavements have made the anatomically distinct muscle compartments less informative. Recently, there was an attempt to quantify the activation patterns of extensor digitorum communis compartments by carefully placing individual surface EMG electrodes based on the anatomical compartments obtained through cadaver data, and it was concluded that the recorded cross-talk from neighboring compartments can reach over 53% (which is the amount of EMG recorded from the neighboring electrode relative to the EMG recorded directly above the target muscle; Leijnse et al., 2008a).

The selective activation of extensor digitorum communis has been studied through the analysis of finger kinematics and end-point force production in both human subjects and animal models (Häger-Ross and Schieber, 2000; Zatsiorsky et al., 2000; Lang and Schieber, 2003; Schieber and Santello, 2004), and through the analysis of motor unit recruitment and discharge synchrony patterns (Keen and Fuglevand, 2004a,b; van Duinen et al., 2009). Our results in the current study extend previous findings and provide information about the global spatial activation patterns of the extensor muscle. Specifically, we found that the index and middle fingers have the most distinct activation patterns compared with other finger extensions. This finding is consistent with the enslaving of finger kinematics and fingertip force production results (Häger-Ross and Schieber, 2000; Zatsiorsky et al., 2000), which show that the index and middle fingers have a greater degree of individuation in comparison with the little and ring fingers. The less distinctive muscle activation patterns during little and ring finger extension can arise from the higher level of common drive across compartments as quantified by the motor unit firing synchrony (Keen and Fuglevand, 2004a). In addition, the compartments controlling little and ring finger extension are smaller and are anatomically in proximity to each other compared with the index and middle finger compartments, and only a nine-electrode band was used to record the extensor and flexor muscles.

It is possible that the EMG electrode array utilized in this study does not have the resolution (see further discussion in the Limitations of the Study Section) to capture the distinct activation patterns of the little and ring finger compartments and other small changes in the activation patterns between tasks. However, using a 16 × 8 grid (with a fixed 8 mm electrode spacing) to cover just the extensor digitorum communis muscle in a preliminary study, we found that the overall activation pattern is still not distinct between little and ring finger extensions. A recent study, using 128 grid electrodes, also reported similar activation patterns during little and ring finger extensions (Gallina and Botter, 2013). Therefore, it is unlikely that the similar activation patterns arose from a recording grid with a large inter-electrode distance.

In agreement with an earlier study using high-density grid electrodes (Gallina and Botter, 2013), we also found that the activation regions are organized more distinctively in the proximal–distal direction rather than in the radial–ulnar direction that has been found in cadaver studies (von Schroeder and Botte, 1995; Leijnse et al., 2008b). Such radial–ulnar organization has also been used for guiding needle placement in intramuscular recordings (Keen and Fuglevand, 2004b; van Duinen et al., 2009; Birdwell et al., 2013). The potential discrepancy (the radial–ulnar organization in cadaver studies vs. the proximal–distal organization in EMG recordings) can come from the fact that the extensor muscle is a cylindrical muscle that orients along the forearm, and the different compartments are not running parallel but have fascicles overlap obliquely between compartments (Leijnse et al., 2008b). Therefore, the resolution of localizing the different activation regions is higher in the proximal–distal direction.

In addition, the radial to ulnar organization order of the different compartments found in our study was ring, index or little, and middle fingers. In contrast, the organization based on a cadaver study (Leijnse et al., 2008b) followed an order of index, middle, ring, and little fingers. The different ordering can partly come from the different arm configurations. Namely, the forearm was pronated 90° in our experiment, but was supinated 90° in the cadaver study. The arm configuration differences can shift the order of the distal tendons and some distal portion of the extensor muscle around the axis of the forearm circumference, therefore leading to different ordering effect. This order shift can also come from low level co-activation of undesired compartments of the extensor digitorum communis or even flexor muscles. Especially in ring finger activation, a high enslaving effect has been reported (van Duinen et al., 2009).

### Influence of effort level and muscle constraint

When the subjects extended their fingers at different effort (or muscle contraction) levels and at different finger constraint conditions (static vs. dynamic), the muscle activation map was relatively consistent across conditions, and generally, < 10% of difference was observed in the SSD calculations (Figure 5). It is possible that the electrode configurations used in the current study was not dense enough to detect the muscle activation shifts (see further discussion in the Limitations of the Study Section), and a more densely distributed electrode array than our current electrode configuration may help to better capture the potentially altered activation patterns across different tasks. However, the system was able to capture a small but consistent shift of the activation centroid (Figure 4A) at high contractions levels as well as at dynamic contractions.

The shift toward the proximal tendon may be largely due to a larger degree of shortening of the muscle belly together with a lengthening of distal tendons during higher muscle contraction levels and especially during high effort dynamic contractions. The centroid shift of the middle finger activation toward the distal tendon can partly arise from a lengthening of the proximal tendons and a shortening of the muscle belly, because the middle finger centroid is located at the very proximal end of the extensor digitorum communis, and stretch of the distal tendon had little effect on the centroid shift. In addition, an earlier study has shown that the enslaving effect (recruitment of motor units in undesired compartments) increases with force level (van Duinen et al., 2009). Thus, an increased co-activation of multiple compartments can also contribute to the centroid shift at high contraction levels. Lastly, a possible non-uniform distribution of the motor units with different recruitment thresholds can contribute to such small shifts of activation regions. The non-uniform distribution of motor units in proximal–distal direction has been observed in biceps brachii and rectus femoris (Jennekens et al., 1971; Holtermann et al., 2005).

### Limitations of the study

One focus of our current study was to investigate the potential influence of low level co-activations of different muscle compartments of the extensor digitorum communis and other muscles, including finger flexors, on motor output. We placed electrodes over the entire forearm, covering both extensors and flexors, in order to record any possible muscle activations. However, due to the channel limitations of our recording system, coverage of the entire arm resulted in inter-electrode spacing that was relatively large (approximately in the range of 17–30 mm). This low resolution recording (with spatial sampling below the spatial Nyquist rate) could distort our RMS map estimations across different task conditions, and, therefore, bias the estimated centroids and SSD-values in our results.

### Implications

The information obtained from the global spatial activation map of the extensor muscle has several potential implications. First, in combination with the anatomical features of the different compartments, the selective activation map of the extensor digitorum communis can be used as a guidance for theoretical research that investigates the focalized activation of the extensor compartments. Previous studies have examined the voluntary recruitment and discharge patterns of motor units across compartments (Keen and Fuglevand, 2004a; van Duinen et al., 2009) as well as activation patterns through intraneural micro-stimulations (Keen and Fuglevand, 2004b). However, the electrode insertion is primarily based on the anatomical radial–ulnar arrangement of the extensor muscle. Our current study shows that the compartment organizations are more distinct in the proximal–distal direction than in the radial–ulnar direction. Such information can help us better localize the muscle compartments and yield more accurate information regarding activation interactions between compartments.

Second, localizing the specific muscle activation regions can also be beneficial for applied research. For example, the finger-specific neural information can facilitate the natural control of motorized prosthetic hands with multiple controllable degrees of freedom (Birdwell et al., 2013), and the spatial feature information of the muscle activation can also be used to improve the robustness of the prosthetic control (Stango et al., 2014). Similarly, the spatial activation pattern can also be used as a triggering source to facilitate individuated finger motion in individuals with neuromuscular disorders (Maneski et al., 2013; Taheri et al., 2014).

## Conclusions

Using HD EMG recordings, we quantified the global spatial activation patterns of the entire extensor digitorum communis muscle in individuated finger extensions with different muscle contraction levels and muscle constraint conditions. The spatial activation information provides means of localizing the finger-specific compartments of the extensor digitorum communis. However, additional investigation, potentially at the motor unit pool level, is needed to better understand the neural activation strategies of the multi-compartment muscle.

## Conflict of interest statement

The authors declare that the research was conducted in the absence of any commercial or financial relationships that could be construed as a potential conflict of interest.
